# Draft Genome of Pseudomonas sp. Strain 11/12A, Isolated from Lake Washington Sediment

**DOI:** 10.1128/genomeA.01587-14

**Published:** 2015-02-19

**Authors:** Tami L. McTaggart, Nicole Shapiro, Tanja Woyke, Ludmila Chistoserdova

**Affiliations:** a Department of Chemical Engineering, University of Washington, Seattle, Washington, USA; b DOE Joint Genome Institute, Walnut Creek, California, USA

## Abstract

We announce here the genome sequencing of Pseudomonas sp. strain 11/12A from Lake Washington sediment. From the genome content, a versatile lifestyle is predicted but not one of bona fide methylotrophy. With the availability of its genomic sequence, Pseudomonas sp. 11/12A presents a prospective model for studying microbial communities in lake sediments.

## GENOME ANNOUNCEMENT

When natural microbial communities from Lake Washington are incubated under the atmosphere of methane, simple and semistable communities are formed consisting of bona fide methanotroph species and of nonmethanotrophic satellite species. Some of the types found to persist in such methane-fed microcosms are the Pseudomonas species (1). Pseudomonas sp. strain 11/12A was isolated from such an enrichment culture that was incubated at 10°C in a minimal salts medium, with multiple transfers and dilutions, for approximately 18 months (1), by plating onto nutrient broth (NB) agar medium (Difco). Axenic culture of Pseudomonas sp. 11/12A was obtained by selecting a single colony, followed by restreaking multiple times onto fresh NB plates.

The draft genome of Pseudomonas sp. 11/12A was generated at the Department of Energy (DOE) Joint Genome Institute (JGI), Walnut Creek, CA, USA, using the Pacific Biosciences (PacBio) sequencing technology (2). All general aspects of library construction and sequencing performed at the JGI can be found online (see http://www.jgi.doe.gov). The raw reads were assembled using HGAP (version 2.2.0.p1) (3). The final draft assembly contains 2 contigs in 2 scaffolds, totaling 6,778,451 bp in size. Genes were identified using Prodigal (4), followed by a round of manual curation using GenePRIMP (5). The predicted coding sequences (CDSs) were translated and used to search the National Center for Biotechnology Information (NCBI) nonredundant database, UniProt, TIGRFam, Pfam, KEGG, COG, and InterPro databases. The tRNAscan-SE tool (6) was used to find tRNA genes, whereas rRNA genes were found by searches against models of the rRNA genes built from SILVA (7). Other noncoding RNAs, such as the RNA components of the protein secretion complex and RNase P, were identified by searching the genome for the corresponding Rfam profiles using Infernal (http://infernal.janelia.org). Additional gene prediction analysis and manual functional annotation were performed within the Integrated Microbial Genomes and Metagenomes (IMG) platform (http://img.jgi.doe.gov) developed by the JGI (8).

From the genome content, a versatile lifestyle can be predicted for Pseudomonas sp. 11/12A, including some of the pathways for single-carbon (C_1_) metabolism. A gene cluster is present encoding proteins showing homology with the proteins for the *N*-methylglutamate pathway for methylamine oxidation (MgdABCD, Gma, and MgsABC) (9), along with genes encoding tetrahydrofolate-linked C_1_ transfer reactions. A gene encoding formaldehyde dehydrogenase is also present. However, no traditional pathways for C_1_ assimilation (10) have been identified. The availability of this genomic sequence makes Pseudomonas sp. 11/12A a prospective model for studying microbial communities in lake sediments.

### Nucleotide sequence accession number.

The genome sequence has been deposited in GenBank under the accession no. JUGV01000001.
